# Stress granules and organelles: coordinating cellular responses in health and disease

**DOI:** 10.1093/procel/pwae057

**Published:** 2024-10-23

**Authors:** Ying Liu, Yin Li, Peipei Zhang

**Affiliations:** Department of Biochemistry and Molecular Biology, School of Basic Medical Sciences, Peking University Health Science Center, Beijing 100191, China; Department of Biochemistry and Molecular Biology, School of Basic Medical Sciences, Peking University Health Science Center, Beijing 100191, China; Department of Biochemistry and Molecular Biology, School of Basic Medical Sciences, Peking University Health Science Center, Beijing 100191, China; Key Laboratory for Neuroscience, Ministry of Education/National Health and Family Planning Commission, Peking University, Beijing 100083, China

**Keywords:** stress granules, membraneless organelle, organelles, interplay, techniques

## Abstract

Membrane-bound organelles and membraneless organelles (MLOs) coordinate various biological processes within eukaryotic cells. Among these, stress granules (SGs) are significant cytoplasmic MLOs that form in response to cellular stress, exhibiting liquid-like properties alongside stable substructures. SGs interact with diverse organelles, thereby influencing cellular pathways that are critical in both health and disease contexts. This review discusses the interplay between SGs and organelles and explores the methodologies employed to analyze interactions between SGs and other MLOs. Furthermore, it highlights the pivotal roles SGs play in regulating cellular responses and the pathogenesis of amyotrophic lateral sclerosis. Gaining insights into these interactions is essential for deciphering the mechanisms underlying both physiological processes and pathological conditions.

## Introduction

Throughout the life cycle of eukaryotic cells, a variety of biological processes occur in a coordinated manner. Cell compartmentalization plays a crucial role in ensuring precise control over the timing and spatial distribution of these processes (Banani et al., 2016). Cell compartmentalization depends on the formation of distinct “rooms” within cells, known as organelles in eukaryotic cells, which include membrane-bound organelles and membraneless organelles (MLOs).

MLOs take the form of macromolecular condensates, often composed of proteins and nucleic acids (DNA/RNA), and exhibit diverse morphology (Hirose et al., 2022). Liquid–liquid phase separation (LLPS) is a crucial physical process involved in the formation of many MLOs. This process occurs when the concentration and identity of macromolecules, along with environmental factors such as temperature, salt composition, and pH, reach conditions where homogeneity cannot be maintained within the cytoplasm or nucleoplasm (Alberti et al., 2019). However, since some MLOs consist of distinct subcompartments with varying physical characteristics, such as nucleolus (Banani et al., 2017), the formation of MLOs may involve not only LLPS but also liquid-to-solid phase transitions (Gomes and Shorter, 2019). Consequently, different MLOs can be liquid-like (Brangwynne et al., 2009), gel-like (Riback et al., 2017), and solid-like (Cereghetti et al., 2021). Moreover, further studies have illustrated that phase separation coupled with percolation (PSPC) can enhance our understanding of the principles behind different condensate formations (Mittag and Pappu, 2022).

Canonical MLOs include cytoplasmic MLOs such as stress granules (SGs), processing bodies (PBs), and numerous nuclear MLOs, such as paraspeckles, Cajal bodies, nucleoli, and PML nuclear bodies (PML NBs) (Alberti and Hyman, 2021). In addition, there are novel biological condensates with distinct biological functions and localizations, such as mesh-like TIS granules formed based on RNA-binding protein TIS11B (Ma and Mayr, 2018), cytosolic phase-separated DIAPH3 granules (Zhang et al., 2023), and cancer-associated unconventionally MLOs formed by ALK fusion oncoproteins (Tulpule et al., 2021). Furthermore, many novel MLOs with unknown functions remain to be characterized (Li et al., 2024).

SGs are important cytoplasmic ribonucleoprotein (RNP) granules, formed through RNA–protein, RNA–RNA, and protein–protein interactions (Ripin and Parker, 2023). They are dynamically assembled in response to various stressors, which is correlated with global translation inhibition. While they exhibit liquid-like behaviors, they also contain stable substructures (Protter and Parker, 2016). SGs play roles in various physiological and pathological processes, including microbial infection (Paget et al., 2023), apoptosis (Arimoto et al., 2008), and tumor development (Li et al., 2023). However, SGs are notably associated with neurodegenerative diseases, including Alzheimer’s disease (Apicco et al., 2018), amyotrophic lateral sclerosis, frontotemporal dementia (FTD) (Fang et al., 2023), Parkinson’s disease (Repici et al., 2019), and Charcot–Marie–Tooth diseases (Cui et al., 2023).

Despite extensive researches into the role of SGs and their relationship with various diseases, many questions remain unanswered. Key areas needing further investigation include how SGs are involved in translation control and RNA metabolism, as indicated by gene ontology (GO) analyses of the SGs proteome (Millar et al., 2023). These functions are intricately linked to the pathogenesis of neurodegenerative diseases (Storkebaum et al., 2023). Therefore, more researches are necessary to fully understand the complex roles played by SGs.

Recent reviews have shown that RNP granules can interact with each other to globally participate in RNA metabolism, such as RNA trafficking, splicing, and decay (An et al., 2021). In addition, studies have revealed that MLOs can interact with membrane-bound organelles to facilitate their assembly and trafficking (Zhao and Zhang, 2020). These studies suggest that the connection between SGs and other organelles could enhance our understanding of SGs' functions in both physiological and pathological processes.

In our review, we summarized the interactions between SGs and various organelles and outline methods that may assist in studying these interactions. Furthermore, we underscored the importance of these interactions to better understand the multifaceted roles of SGs.

## Stress granule

### Introduction to stress granule

SGs are typical RNP granules composed of proteins and RNAs. Most proteins within SGs are RNA-binding proteins (RBPs) enriched with domains or motifs that contribute to phase separation, such as RasGAP SH3-binding protein (G3BP) and DEAD-box ATPases (Millar et al., 2023). In addition, SGs also contain proteins of translationally arrested pre-initiation complexes, such as the 40S subunits (Kedersha et al., 2002). This is correlated with the characteristic that the formation of SGs is associated with translational inhibition. Consequently, drugs that stabilize translation polysomes can inhibit the formation of SGs, such as emetine, while drugs that promote the disassembly of polysomes can promote their formation, such as puromycin (Kedersha et al., 2000). RNAs within SGs include mRNAs associated with global transcriptome and some antisense noncoding RNAs (Khong et al., 2017). Recent studies showed that whether RNAs can enter SGs is correlated to their transcript lengths, nucleobase modifications, and base ratios (Ren et al., 2023; Van Treeck et al., 2018).

SGs form in response to different stress conditions, including biological stressors like hypoxia and viral infections, environmental changes, such as temperature fluctuations and osmotic pressure shifts, and exposure to specific chemicals like sodium arsenite (Wolozin and Ivanov, 2019). SG formation often occurs due to the stalling of translation initiation, which can be triggered by the phosphorylation of eIF2α—a crucial protein involved in translation initiation (Hofmann et al., 2021). However, SGs can also form independently of phosphorylated eIF2α, such as glucose starvation-induced SGs in fission yeast (Nilsson and Sunnerhagen, 2011), and SGs formed after treatment with eIF4A inhibitor or exposure to hyperosmotic conditions (Aulas et al., 2017).

### Stress granule and neurodegenerative diseases

SGs are notably associated with many neurodegenerative diseases, such as Alzheimer’s disease (Apicco et al., 2018), Huntington’s disease (Sanchez et al., 2021), Parkinson’s disease (Repici et al., 2019), amyotrophic lateral sclerosis (ALS), and FTD (Fang et al., 2023). Mutations in many SGs' components, such as TDP-43 (Sreedharan et al., 2008) and FUS (Kwiatkowski et al., 2009) have been identified in patients with these diseases (Li et al., 2022; Wolozin and Ivanov, 2019). These phase-separated proteins with diseases associated mutation, such as TDP-43 (Liu-Yesucevitz et al., 2010), FUS (Shelkovnikova et al., 2014a), Tau (Younas et al., 2020), and Huntingtin (Ratovitski et al., 2012) are found to form aggregates in cytoplasm that can colocalize with SGs in the brain of patients or SGs formed after specific stress treatments. In addition, researchers have recently found that dysfunction in SGs also contributes to the pathogenesis of Charcot–Marie–Tooth type 2 neuropathies (Cui et al., 2023).

These researches suggest that the manipulation of SGs dynamics and the proteins involved could be potential therapeutic targets for these diseases. Understanding the precise mechanisms by which SGs contribute to neurodegeneration is essential for developing new strategies to mitigate or prevent the progression of these debilitating conditions. For instance, targeting the pathways that regulate SGs assembly and disassembly, or modulating the interactions between SGs and other cellular components, could offer new avenues for therapeutic intervention.

Moreover, the role of SGs in neurodegenerative diseases underscores the importance of further researches into their formation, composition, and function. Investigating how SGs interact with other cellular organelles and how these interactions are altered in disease states could provide valuable insights into the cellular mechanisms underlying neurodegeneration. In addition, exploring the potential for SGs to serve as biomarkers for early diagnosis and progression of neurodegenerative diseases could have significant clinical implications. Continued researches into the biology of SGs and their interactions with other cellular components are essential for developing new therapeutic strategies and improving our understanding of neurodegenerative disease mechanisms.

## Interaction between stress granule and other organelles

**Figure 1.**
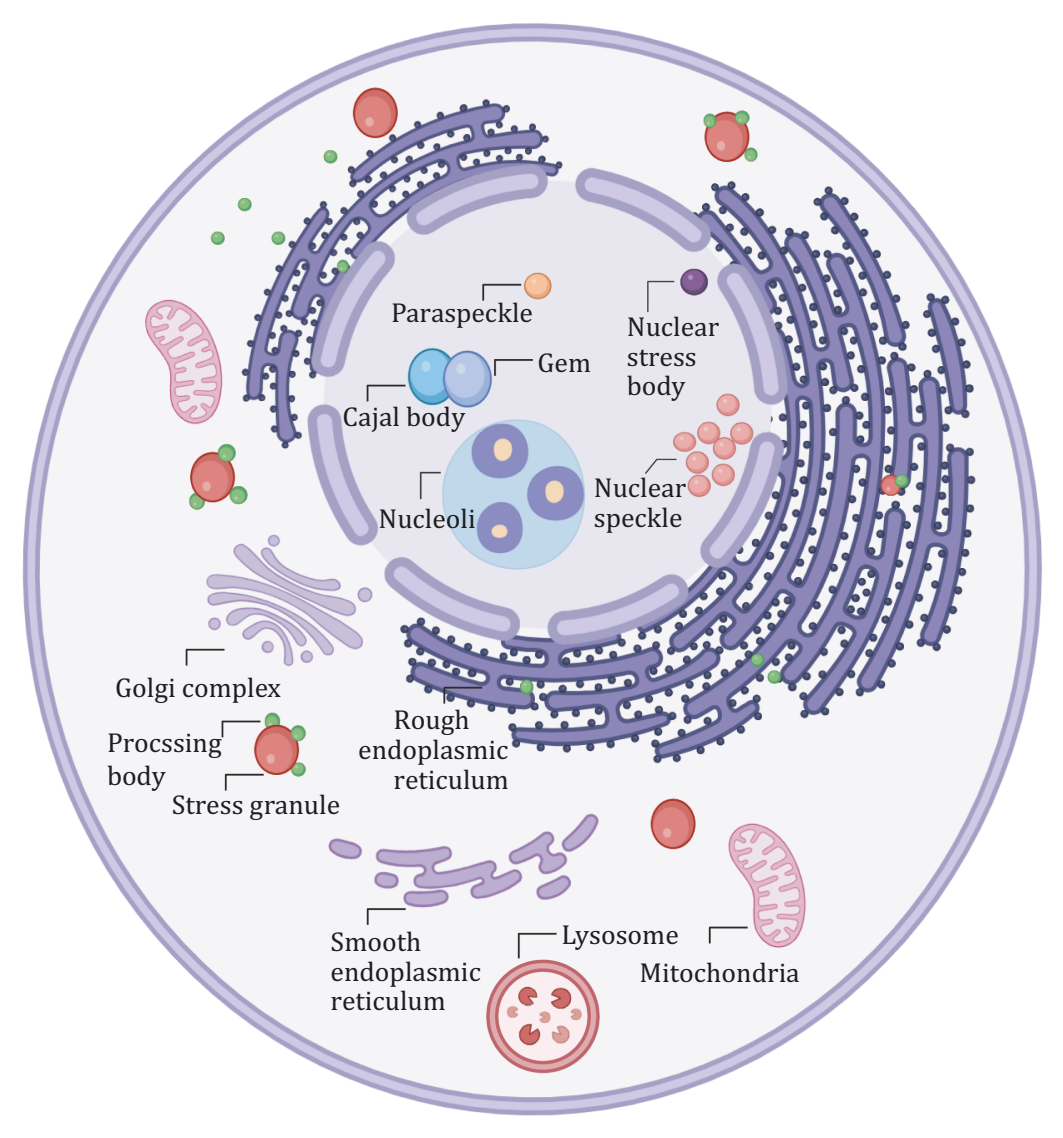
Membrane-bound and membraneless organelles that engage in crosstalk with SGs. SGs can interact with various organelles under different stress conditions to promote cellular responses to these stresses (created with BioRender.com, with permission).

As previously mentioned, the crosstalk between SGs and other organelles can enhance our understanding of SGs’ functions, further elucidating why neurodegenerative diseases, such as ALS and FTD, are associated with SGs. Recent researches have indicated that SGs can interact with both MLOs, such as PBs and paraspeckles, and membrane-bound organelles, such as lysosomes and the endoplasmic reticulum (ER) (Fig. 1). In this section, we will specifically illustrate the direct physical interactions and indirect interactions between SGs and other organelles, including MLOs and membrane-bound organelles.

### Interaction between stress granule and other membraneless organelles

#### Stress granule and processing body

PBs have long been studied to be associated with SGs. PBs are consistently present in the cytoplasm of various cell lines containing proteins that function in mRNA decapping, decay, and miRNA-mediated silencing (Hubstenberger et al., 2017). Both SGs and PBs can be influenced by various stimuli. The number of PBs increases after exposure to arsenite, but heat shock does not seem to affect their quantity (Kedersha et al., 2005). SGs induced by arsenite exposure often colocalize with PBs (Ivanov et al., 2019). Researchers have also identified proteins that regulate the physical interaction between these two MLOs. For instance, TTP, an mRNA decay activator protein, can facilitate the fusion of SGs and PBs (Kedersha et al., 2005). In addition, overexpression of CPEB1, a cytoplasmic polyadenylation element-binding protein, can induce SG assembly and subsequently recruit PBs to associate with SGs (Wilczynska et al., 2005). Recent studies have demonstrated that knocking down or eliminating critical components of PBs, such as the RNA helicase DDX6 or its interactors like CNOT1 and 4E-T, can lead to the formation of hybrid PB/SG granules. The alteration in PBs' composition and function can subsequently increase the number of SGs after exposure to sodium arsenite (Majerciak et al., 2023). Furthermore, recent research has also shown that UBAP2L, a core component of SGs, can localize to both SGs and PBs and further regulate the physical interaction between these two MLOs (Riggs et al., 2024). These proteins that can regulate both SGs and PBs are identified as shared components of these two MLOs (Youn et al., 2019). This discovery suggests that researchers can uncover insights into the interaction between different MLOs by examining the overlapping components of these two organelles. Moreover, there is evidence of mRNA shuttling bidirectionally between these two organelles in mammalian cells, although the exact biological significance of this phenomenon remains to be fully elucidated (Moon et al., 2019). However, studies in yeast have revealed the indispensable role of RNAs transiting from PBs to SGs in SGs formation, where the release of RNAs from PBs is regulated by the ATPase activity of Dhh1, a member of the DEAD-box ATPase family (Hondele et al., 2019).

The movement of proteins and RNAs between SGs and PBs is of critical importance. As previously discussed, numerous studies have demonstrated that PBs and SGs can physically interact with one another or form hybrid granules. However, it has also been observed that they can exist in separate entities, leading to the question of what factors regulate these distinct forms. Extensive research has elucidated these phenomena from a biophysical perspective. This research suggests that the formation and maintenance of MLOs is context-dependent, governed by the interactions among various RBPs and their associations with RNAs. According to this theory, the core proteins that are crucial for the formation of SGs and PBs can be viewed as nodes that may compete for shared components of both granules (Sanders et al., 2020). Thus, it becomes more comprehensible why SGs and PBs can interact to varying degrees. Furthermore, this model can further promote our understanding of other physical interactions between other MLOs.

#### Stress granule and neuronal transport granule

Neuronal transport granules are RNPs that dynamically move along microtubules of dendrites and axons, primarily functioning to spatiotemporally control the distribution and translation of RNAs. The components of neuronal transport granules include mRNAs and various translation-associated proteins, such as ribosome protein and elongation factors, and other RBPs (Fernandopulle et al., 2021). They also share many components with SGs, such as Fragile X mental retardation protein (Zalfa et al., 2006) and DEAD-box 3 helicase (Elvira et al., 2006). Furthermore, researchers have found that the marker of neuronal transport granules, Staufen proteins, and mammalian Pumilio2 can relocate to SGs following sodium arsenite treatment (Vessey et al., 2006). A recent study has also shown that deubiquitylating enzyme OUTD4, which normally localizes to neuronal transport granules, can be recruited to SGs after sodium arsenite treatment, thereby regulating the dynamics of SGs (Das et al., 2019). However, the significance of the exchange of substances between neuronal transport granules and SGs remains to be further elucidated. Maybe in neurodegenerative disease, mutations of core components of SGs, such as TIA1 in ALS, can impair the dynamics of SGs (Mackenzie et al., 2017), preventing components from other MLOs, such as neuronal transport granules, from returning to their original locations and recovering their functions. Furthermore, it is noteworthy that the marker of SG, G3BP1, is also capable of forming SG-like aggregates in axon of neurons to regulate axonal mRNA translation and axonal growth (Sahoo et al., 2018), and they can also travel along microtubule by hitchhiking lysosome (Liao et al., 2019). This suggests that these granules could potentially be categorized as neuronal transport granules. However, it is important to distinguish them from SGs that form as a response to cellular stress, as their composition, function, and formation mechanisms may differ significantly.

#### Stress granule and nuclear membraneless organelles

Except direct physical interaction between SGs and PBs, indirect interaction exists between SGs and nuclear MLOs. Recent researches also showed interaction between SGs and PML NBs. PML NBs are dynamic MLOs enriched with PML proteins, forming a shell-like structure. The classic post-translational modification, SUMOylation, plays a crucial role in the biogenesis and regulation of PML NBs through interactions with SUMO-SIM (SUMO interaction motif) (Lallemand-Breitenbach and De Thé, 2018). Consequently, PML NBs serve as a crucible for protein SUMOylation, contributing to the clearance of intranuclear inclusion bodies associated with various neurological diseases through SUMO-dependent ubiquitination (Freemont, 2000; Woulfe, 2008). The relationship built between PML NBs and SGs is also built upon SUMO-dependent ubiquitin. Unfolded proteins produced after heat shock or oxidative stress can be partially cleared by the SUMO-targeted E3 ubiquitin ligase (StUbL) pathway (Gärtner and Muller, 2014). Moreover, Jan Keiten-Schmitz (2020) and their research team discovered that impairments in the StUbL pathway led to the delayed disassembly of SGs, strongly suggesting a connection between PML NBs and SGs. Subsequently, Francesco Antoniani and their colleagues delved deeper into the interplay between PML NBs and SGs. Their research unveiled a reduction in PML NBs in the frontal cortex and hippocampus of certain ALS-FTD patients. Mimicking this reduction by depleting PML or Ubc9 resulted in delayed SG disassembly and the altered localization of defective ribosomal products. Some of these defective products shifted from the nucleus to cytoplasmic SGs, ultimately hindering SG dynamics (Antoniani et al., 2023).

Besides PML NBs, recent research has found reciprocal regulation between paraspeckles and SGs. Paraspeckles are characterized by their scaffold, a long noncoding RNA (LncRNA) known as nuclear paraspeckle assembly transcript 1 (NEAT1). These structures also contain a multitude of RBPs, including members of the drosophila behavior human splicing (DBHS) family like SFPQ, NONO, and PSPC1 (Wang and Chen, 2020). Paraspeckles are observed in a wide range of cell lines (Hirose et al., 2022), typically numbering around 5–20 per nucleus. However, this count can escalate in response to cellular stressors such as heat shock and hypoxia (McCluggage and Fox, 2021). Certain stimuli that trigger the formation of SGs, such as sodium arsenite and MG132, have also been shown to promote an increase in the number of paraspeckles. Intriguingly, when the formation of SGs is inhibited, the concurrent rise in paraspeckle numbers is impaired. Researchers have proposed an explanation for this phenomenon: SGs might serve as sites for sequestering negative regulators of paraspeckles, such as UBAP2L and YBX125. This interaction suggests a complex interplay between these two distinct MLOs, possibly contributing to the orchestration of cellular responses to stress (An et al., 2019).

Except PML NBs and paraspeckles, recent studies have also highlighted the interaction between SGs and Cajal bodies and Gems. Cajal bodies and Gems are two nuclear MLOs with similar sizes and shared components, such as SMN (Nizami et al., 2010). The post-translational modification of SMN can regulate whether Gem can dock on Cajal bodies (Courchaine et al., 2021). Further investigations have shown that treating cells with sodium arsenite or thapsigargin leads to the inhibition of Cajal bodies and Gems formation. This inhibition may stem from stress-induced interference with UsnRNP (U small nuclear RNP) traveling into the nucleus, which serves as materials for the formation of Cajal bodies and Gems, as SGs can block importin within their structure (Rossi et al., 2020). This phenomenon may partially explain the reduced Gems observed in patients with ALS and spinal muscular atrophy (Staněk and Fox, 2017).

Moreover, recent research has discovered that the assembly of SGs can be regulated by nuclear RNA processes, such as transcription and splicing inhibition, which can prevent the formation of SGs. These nuclear RNA processes influence the levels of cytoplasmic RNAs, thereby affecting the assembly of SGs (Angel et al., 2024). Since many nuclear MLOs, like nuclear speckles, are involved in RNA processing and splicing (Galganski et al., 2017), understanding their role may shed light on how nuclear MLOs regulate SGs. Furthermore, some nuclear-cytoplasmic transport factors, such as Importins and Exportin-1, are recruited into SGs under certain stress conditions, suggesting that SGs might also impact the function and composition of nuclear MLOs (Zhang et al., 2018).

### Interaction between stress granule and membrane-bound organelles

#### Stress granule and lysosome

Recent studies have highlighted the interaction between SGs and lysosomes, particularly focusing on lysosome damage. Jia et al. discovered that treatment with Leu-Leu-O-Me (LLOMe), a substrate of cathepsin C known to induce lysosome damage (Papadopoulos et al., 2020), triggers the formation of SGs in an eIF2α-dependent manner. Moreover, they demonstrated that the recruitment of G3BP1/NUFIP2 to lysosome damage relies on the Atg8ylation of the lysosome membrane following damage. The function of G3BP1/NUFIP2 recruitment involves the inactivation of the mTOR complex (Jia et al., 2022). Subsequent mTOR inactivation leads to TFEB dephosphorylation, thereby regulating lysophagy and lysosome biogenesis (Settembre et al., 2013). In addition, Bussi et al. (2023) found that SGs directly contribute to stabilizing lysosome membranes by localizing at the pores of damaged areas, essentially acting as plugs. This phenomenon is explained by the wetting of SGs, which aids in the passive sealing of pores.

In addition to lysosome damage, recent research has revealed that the interaction between SGs and lysosomes is mediated by the bridge protein Annexin A11. Due to its unique properties, Annexin A11 possesses a low-complexity domain at its N-terminus and a membrane-binding domain at its C-terminus. This distinctive composition enables Annexin A11 to act as a linker between lysosomes and SGs. Furthermore, studies have demonstrated that in nonstressed neurons, RNA granules marked by SG components, such as G3BP1 and Caprin1, can travel along microtubules in association with lysosomes, with Annexin A11 serving as a pivotal mediator to facilitate long-range RNA transport. Researchers also showed that ALS-associated mutations in Annexin A11 disrupt RNA granule traveling (Liao et al., 2019). It is noteworthy that in Liao’s study, they also illustrated that TDP-43 labeled RNA granules can travel with lysosome along the axon and interact with Annexin A11, and ALS-associated mutations of Annexin A11 disrupt this interaction with TDP-43. This finding is particularly intriguing, as recent studies have shown the colocalization of Annexin A11 aggregates with TDP-43 inclusions in cases of frontotemporal lobar degeneration with TDP-43 inclusions (FTLD-TDP) type C (Robinson et al., 2024). Furthermore, a recent study has delved deeper into the structural composition of these inclusions, revealing that the N-terminus of Annexin A11, which is its low-complexity domain, is the region that aggregates within the amyloid filaments of the inclusions. The same study also identified C-terminal truncations of Annexin A11 in patients with FTLD-TDP type C, suggesting a loss of function of Annexin A11 (Arseni et al., 2024). As mentioned before, Liao’s research indicates that the membrane-binding domain of Annexin A11 is located at its C-terminus. Therefore, the dissociation between the N- and C-terminus of Annexin A11 could impair its role in RNA granule transport, potentially contributing to the pathogenesis of FTLD-TDP type C.

In addition, it is fascinating to observe that other neuronal RNA granules interact with late endosomes and travel along the microtubules of the axon. These RNA granules associated with endosomes, have the capacity to engage with ribosomal proteins, thereby facilitating local translation of mitochondrial proteins. This localized translation is a key regulatory mechanism that ensures the maintenance of mitochondrial integrity, which is crucial for the overall health and functionality of the neuron (Cioni et al., 2019).

#### Stress granule and endoplasmic reticulum

Regarding the interaction between SGs and the ER, it has been observed that ER stressors can induce the formation of SGs in an eIF2α-dependent manner, such as thapsigargin (Sidrauski et al., 2015). In a study conducted by Lee et al., (2020) it showed that the ER actively contributes to the fission of SGs by traversing the “constricted” neck of the SGs. This mechanism may facilitate the disassembly of SGs during the recovery process after exposure to stress. In addition, there is a hypothesis suggesting that the ER might play a role in the assembly of ER-associated SGs, as certain ER-targeted mRNA can be recruited to ER-associated SGs (Child et al., 2021). Recently, researchers also found that the ER transmembrane protein IRE1α can colocalize with SGs during ER stress. This co-localization creates a more efficient workstation for IRE1α, enhancing the splicing of XBP1 mRNA (Liu et al., 2024).

#### Stress granule and mitochondria

The potential interaction between SGs and mitochondria was suggested by Liao et al. (2019), demonstrating partial co-localization of SGs with mitochondria. However, the biological significance of this phenomenon remained unclear until 2021 when Triana Amen discovered that SGs formed after prolonged starvation stress can regulate fatty acid β-oxidation (FAO) by influencing mitochondrial voltage-dependent anion channels (VDACs). VDACs serve as channels for importing fatty acids into mitochondria. In this study, researchers identified a direct interaction between SGs, mitochondria, and lipid droplets. The formation of SGs in this process is associated with a reduction in oxidation damage (Amen and Kaganovich, 2021). Furthermore, recently, Kovacs et al. (2023) discovered that aggregates localized in mitochondria containing the super-aggregator Olalp formed after heat shock are SGs. They also observed that the clearance of these aggregates is linked to protease activity within mitochondria, and the disassembly of SGs is associated with the number of lipid droplets.

#### Stress granule and Golgi complex

Catara et al., (2017) demonstrated that Golgi-localized mono ADP-ribosyltransferase, PARP12, can translocate from the Golgi complex to SGs during stress treatments, such as heat shock and sodium arsenite treatment. This translocation is associated with the inhibition of anterograde membrane traffic. Furthermore, a novel finding indicates that GM130, a constituent of the Golgi complex, is capable of interacting with RNAs and key components of SGs, including FXR1, G3BP1, and PABPC1. This interaction leads to the formation of condensates that play a crucial role in stabilizing Golgi membrane tubules within cells that have not been subjected to stress. Interestingly, when cells are exposed to sodium arsenite, a stress-inducing agent, the assembly of SGs attracts the RNAs and RBPs associated with GM130 into the SGs. This recruitment process disrupts the normal function of GM130, ultimately leading to a compromised Golgi structure and potentially affecting various cellular processes that rely on the integrity of the Golgi apparatus (Zhang and Seemann, 2024). The cross-compartmental translocation between the Golgi complex and SGs exemplifies the complexity of intracellular phase separation and functional dynamic reorganization. These discoveries provide new insights into the regulation of cellular stress responses, membrane trafficking, and structure of membrane-bound organelles.

### Interaction between stress granule and other complexes

In addition to typical MLOs and membrane-bound organelles, researches have indicated that SGs can interact with other complexes. Samir et al. (2019) discovered that SGs and the NLRP3 inflammasome compete for DDX3X molecules to regulate the activation of innate responses and guide subsequent cell-fate decisions under stress conditions. This competitive interaction highlights the complex regulatory mechanisms that SGs participate in, influencing both cellular stress responses and immunity. Moreover, Mallarino et al. (2020) demonstrated that components of the cilium, a microtubule-based organelle, can be recruited to SGs during translation inhibition.

An interaction between SGs and microtubules has been established, with studies revealing that the cytoplasmic mobility of SGs is significantly affected by the integrity of the microtubule network. Specifically, targeting microtubules with the drug nocodazole markedly reduces SG mobility and hinders their disassembly, indicating a crucial role for microtubules in regulating SG dynamics. In contrast, manipulation of actin filaments using drugs such as latrunculin does not impact SG mobility (Nadezhdina et al., 2010). Moreover, microtubules have been shown to influence the size of SGs by aggregation of small cytoplasmic granules into larger structures, a process that can be disrupted by treating cells with the microtubule-targeting drug vinblastine, leading to the formation of smaller and more numerous SGs (Chernov et al., 2009). Furthermore, motor proteins such as Dynein and Kinesin have been implicated in the dynamics of SGs, further emphasizing the role of microtubules in SGs regulation (Loschi et al., 2009; Tsai et al., 2009). In addition, recent research indicates that both microtubules and actin filaments can influence the perinuclear localization of SGs (Böddeker et al., 2023). Additional researches are required to elucidate the complex interactions between SGs and the cytoskeleton.

**Figure 2.**
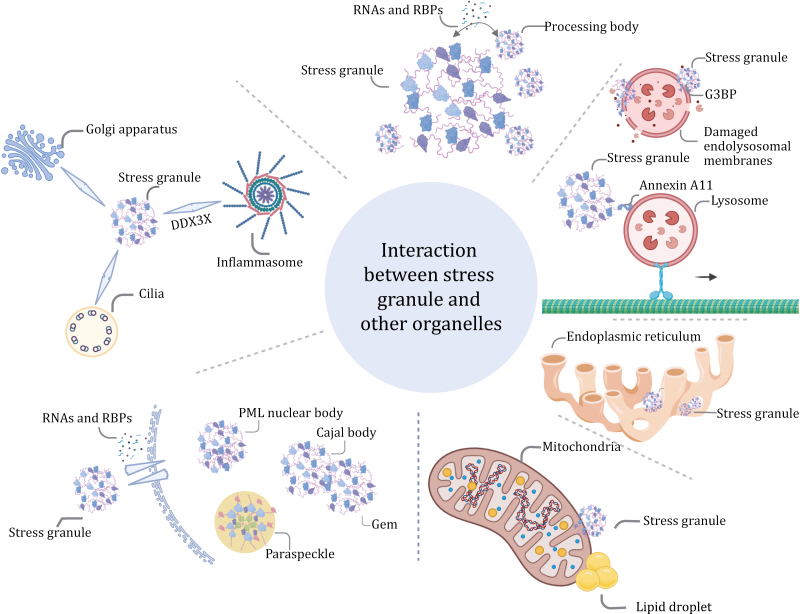
Examples of interactions between SGs and other organelles. SGs can interact with MLOs and membrane-bound organelles through direct physical interaction and indirect crosstalk by exchanging proteins or RNAs (created with BioRender.com, with permission).

In summary, SGs can interact with various organelles under stress conditions, potentially functioning as transfer stations. Components from specific organelles can move into SGs, and once the SGs disassemble, these components may be degraded or return to their original locations (Fig. 2). However, under disease conditions, this crosstalk may be disrupted, potentially leading to dysfunction in different biological processes during recovery from stress. Therefore, understanding the interactions between SGs and other organelles is of great significance.

## Techniques for analyzing interactions between SGs and other MLOs

The examination of the aforementioned content reveals that the interaction among SGs and other MLOs is grounded in their shared components. In addition, it is evident that various MLOs can be influenced by similar stress conditions (Table 1), with many MLOs being concurrently affected in disease model (Table 2). For instance, in the ALS/FTD disease model, arginine-containing dipeptides, generated following the pathogenic expansion of a hexanucleotide repeat (G_4_C_2_) in C9orf72, have been observed to impact the dynamics of SGs, nucleoli, nuclear speckles, and Cajal bodies (Lee et al., 2016), and mutation in TDP-43 and FUS can influence properties of both SGs and other MLOs (Table 2). Furthermore, there are components that shuttle between SGs and other MLOs. For instance, TIAR enters nuclear speckles when cells are treated with anisomycin for 30 min; however, if the treatment time extends to 8 h, TIAR shifts its localization to SGs (Sung et al., 2023). In addition, a key component of paraspeckles, PSPC1, undergoes a change in its localization from paraspeckles to SGs following sodium arsenite treatment (An et al., 2019).

**Table 1.** Examples of stress conditions that can influence membraneless organelles.

Membraneless organelle	Stress conditions	Changes of membraneless organelle
Stress granule	Sodium arsenite, heat shock, MG132, poly(I:C) (An et al., 2019), hyperosmotic stress (sorbitol, sucrose, NaCl), EIF4A inhibitor (Rocaglamide), endoplasmic reticulum stress (Thapsigargin) (Aulas et al., 2017), lysosome-damage induced drug (LLOMe) (Bussi et al., 2023), UV-radiation, mitochondrial poison clotrimazole (Kedersha et al., 2005), and doxorubicin (Zhao et al., 2023)	Stress-induced appearance
Nuclear stress body	Heat shock, amino acid analog azetidine, cadmium sulfate, UV-light (Biamonti and Vourc’h, 2010), serum deprivation, H_2_O_2_, cadmium sulfate, sodium arsenite, and mitoxantrone (Collins et al., 2020)	Stress-induced appearance
A body	Heat shock, acidosis, and transcriptional/proteotoxic stress (actinomycin D and MG132) (Marijan et al., 2019)	Stress-induced appearance
Paraspeckle	Heat shock, hypoxia (Godet et al., 2022), foreign double-strand RNA, proteasome inhibition, and hyperosmotic stress (sorbitol) (An et al., 2019)	Increased in formation
PML nuclear body	Arsenic trioxide treatment (Sahin et al., 2014) and irradiation (Ma et al., 2023)	Increased in formation
	Heat shock and heavy metal stress (Dellaire and Bazett-Jones, 2004)	Increased in fission
Nuclear speckle	Hypoxia (de Oliveira Freitas Machado et al., 2023)	Become dispersed
	Anisomycin (Sung et al., 2023)	Composition changed
	Transcriptional inhibitor (5,6-dichloro-1-β-ribofuranosyl benzimidazole (DRB), α-amanitin, triptolide), heat shock, and heavy metal stress (cadmium) (Kim et al., 2019)	Brighter, rounder, fewer in number, and increased mobility
Processing body	Sodium arsenite, Cycloheximide (Andrei et al., 2005)	Increased in formation
	Cycloheximide (Andrei et al., 2005)	Dissolved
Cajal body	Sodium arsenite, Thapsigargin (Rossi et al., 2020)	Decreased in number
	Bortezomib (Palanca et al., 2014)	Increased in number
	More detailed stress/drugs were reviewed by Boulon et al. (2010)	More detailed changes were reviewed by Boulon et al., (2010)
Nucleolus	Bortezomib; more detailed stress/drugs were reviewed by Boulon et al. (2010)	Increased in number; More detailed changes were reviewed by Boulon et al., (2010)

**Table 2.** Examples of disease-associated proteins that can influence membraneless organelle.

Membraneless organelle	Disease-associated mutations	Model	Changes of membraneless organelle
Stress granule	Toxic arginine-containing dipeptide repeats produced by the expansion of a G_4_C_2_ in C9ORF72 in ALS/FTD (Lee et al., 2016)	HeLa cells	Dynamics impaired
	TDP-43^M337V^ in ALS/FTD (Dubinski et al., 2023)	Mice	Assembly impaired in heat shock and aging
	TDP-43^A315T/ M337V^ in ALS (Ding et al., 2021)	NSC-34 motor neuron-like cells	Disassembly impaired in hyperosmotic treatment
	FUS^P525L^ in ALS (Szewczyk et al., 2023)	Human-induced pluripotent stem cells (hiPSCs)	Increased in size and number, but dynamics impaired
	FUS^R495X^ in ALS (Baron et al., 2013)	HEK293T cells	
	TIA1^p362L/A381T^ in ALS/FTD (Mackenzie et al., 2017)	HeLa cells	Disassembly impaired
	GlyRS^P234KY/L129P^ in CMT2 (Cui et al., 2023)	HeLa cells	Composition changed
Paraspeckle	TDP-43 mutation in ALS (Shelkovnikova et al., 2018)	Spinal neurons and glial cells of ALS patients	Augmented assembly
	C9orf72 mutation in ALS (Shelkovnikova et al., 2018)	Spinal neurons and glial cells of ALS patients	Augmented assembly
	FUS^R522G^ in ALS (Shelkovnikova et al., 2014b)	SH-SY5Y cells; COS7 cells	Compromised formation
Nucleolus	Toxic arginine-containing dipeptide repeats produced by the expansion of a G4C2 in C9ORF72 in ALS/FTD (Lee et al., 2016)	HeLa cells	Dynamics impaired
	C9orf72 mutation in ALS (Aladesuyi Arogundade et al., 2021)	Spinal cord motor neurons of ALS patients	Decreased in size
Processing body	Alpha-synuclein produced after inherited triplication or A53T of the SNCA locus in Parkinson’s disease (Hallacli et al., 2022)	Patient-derived induced neuron (iN)	Decreased in number
	FUS^P525L^ in ALS (Takanashi and Yamaguchi, 2014)	SH-SY5Y cells	Decreased in number
Nuclear speckle	Toxic arginine-containing dipeptide repeats produced by the expansion of a G4C2 in C9ORF72 in ALS/FTD	HeLa cells	Dynamics impaired
	Tau (0N4R)^P301S^ in AD (Lester et al., 2021)	HEK293T cells	Dynamics impaired
Cajal body	Toxic arginine-containing dipeptide repeats produced by the expansion of a G4C2 in C9ORF72 in ALS/FTD (Lee et al., 2016)	HeLa cells	Assembly impaired
	FUS^P525L^ in ALS (Rossi et al., 2020)	HeLa cells	Decreased in number

**Figure 3.**
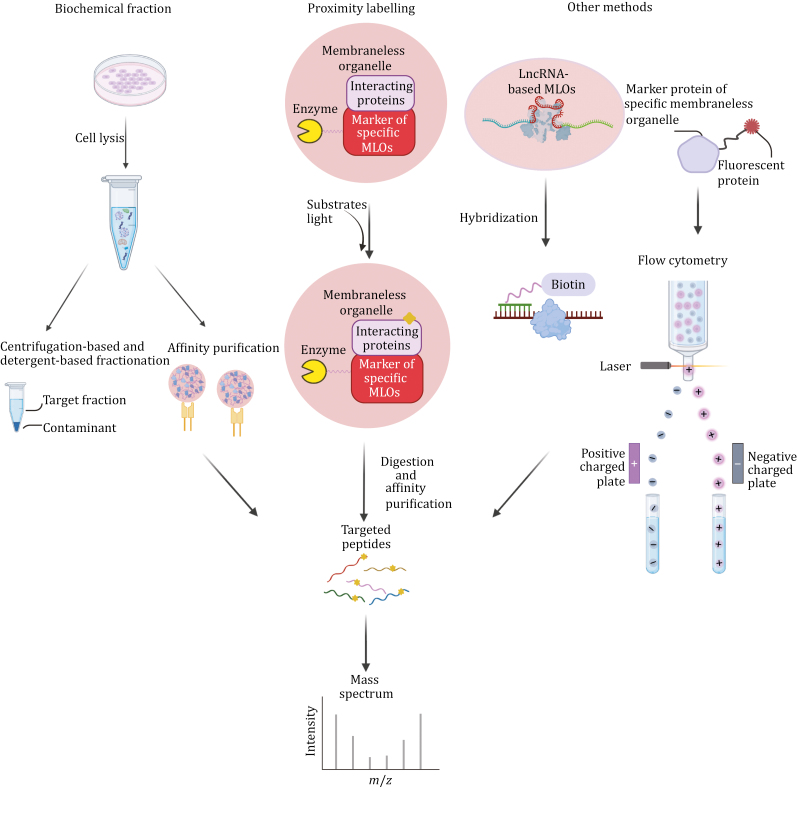
Methods for identifying components of different MLOs. Traditional techniques include biochemical fractionation and proximity labeling. In addition, RNA labeling and flow cytometry methods can be employed for this purpose, offering comprehensive insights into MLO composition (created with BioRender.com, with permission).

The question arises whether other MLOs can also interact with SGs. A valuable approach in investigating this is comparing the shared components of SGs and other MLOs, which can provide significant insights. To identify the overlapping components of specific organelles, it is first necessary to obtain the components of different organelles. Two common strategies for addressing this challenge are biochemical fractionation of MLOs and proximity labeling, both of which rely on mass spectrometry (MS) for component analysis. In addition, there are alternative methods that do not fit into the typical categories of biochemical fractionation or proximity labeling. In the following section, we will provide a comprehensive overview of the utilization, benefits, and limitations of these methods (Fig. 3). And we also summarized approaches applied in acquiring components of some classical MLOs in Table 3. Furthermore, the methods discussed in this section are applicable not only to the study of SGs but also to the investigation of other MLOs. This versatility allows researchers to utilize these techniques to explore the components of newly identified MLOs and to understand their interactions with one another.

**Table 3.** Examples of approaches employed to uncover components of membraneless organelles.

Membraneless organelle	Methods
Stress granule	Biochemical fraction (Jain et al., 2016)
	Proximity labeling: Apex2 (Markmiller et al., 2018) and BioID (Youn et al., 2018)
Processing body	Biochemical fraction (Hubstenberger et al., 2017)
	Proximity labeling: BioID (Youn et al., 2018)
Nuclear speckle	Biochemical fraction (Saitoh et al., 2004)
	Other method: TSA-MS (Dopie et al., 2020) and CHART-MS (West et al., 2014)
Paraspeckle	Biochemical fraction (An et al., 2019)
	Other method: localization screen (Fong et al., 2013; Naganuma et al., 2012); CHART-MS (West et al., 2014)
Nuclear stress body	Other method: ChIRP-MS (Ninomiya et al., 2020)
Nucleolus	Biochemical fraction (Andersen et al., 2005)

### Methods for isolating components of various MLOs

#### Biochemical fraction

Biochemical fractionation is commonly applied to obtain specific subcellular compartments before MS analysis, which includes centrifugation-based fractionation, detergent-based fractionation, electrophoresis, and affinity purification (Christopher et al., 2021). These methods are used to discriminate many MLOs. For example, back to the last century, nucleoli were separated by sucrose density gradient centrifugation (Muramatsu and Onishi, 2008). Then, based on the same principle, researchers from other laboratories also isolated interchromatin granule clusters, which are also called nuclear speckles, by gradient sedimentation (Mintz et al., 1999). However, subcellular compartments discriminated by centrifugation-based fractionation and detergent-based fractionation always bring in contaminants and they cannot be applied to subtly separate MLOs with similar density or solubility. In that case, affinity purification can be more general and useful, as whether we know the critical component, skeleton, or marker of a specific MLO, we can purify it by immunoprecipitation. For example, the core of mammalian SGs is separated by centrifugation-based fraction associated with affinity purification based on their critical marker G3BP1 fused with GFP (Jain et al., 2016). Similar protocols were also applied to separate paraspeckle-like structure (An et al., 2019). Moreover, enriching MLOs, especially those interacting with membrane-bound organelles, can be challenging. Proper biochemical fractionation methods are crucial in such scenarios. Recent advancements have utilized flow cytometry to isolate and purify SGs formed under different stress conditions, followed by MS analysis of their components (Zhou et al., 2024). This method, known as fluorescence-activated particle sorting (FAPS), is adaptable for enriching a range of MLOs. It involves tagging marker proteins with fluorescence and detecting them through flow cytometry platforms. For instance, PB is the first MLO to be enriched using FAPS, followed by a comprehensive analysis of its composition through MS (Hubstenberger et al., 2017). Moreover, FAPS can also be utilized to obtain the components of DACT1 condensates, another noncanonical MLO (Esposito et al., 2021).

However, the procedure of fraction that separates MLOs by mechanical destruction like ultrasonic and centrifugation may drop some materials located at the border of the MLOs, and there may be some components inside MLOs tending to move quickly between MLOs and outside, so other methods need to be developed to remedy these limitations.

#### Proximity labeling

The proximity labeling system comprises three key components, namely the bait protein, the prey protein, and an enzymatic reaction. This system operates by attaching distinctive tags to the prey proteins that come into close proximity to the bait protein, within a defined range of spatial proximity.

The labeling method first applied in living mammalian cells is engineered ascorbate peroxidase (APEX)-based proximity labeling. In this method, APEX is fused to a bait protein, and it tags its neighbors with biotin. This is achieved through the principle that APEX can oxidize biotin-phenol to biotin-phenoxyl radicals in the presence of H_2_O_2_ (Rhee et al., 2013). However, due to the low sensitivity and expression level of APEX, a more sensitive single mutant, APEX2, was subsequently identified. APEX2 shows higher labeling efficiency compared to APEX (Lam et al., 2015). Nevertheless, the use of H_2_O_2_ in the APEX2 method can lead to cytotoxicity, prompting the search for a biologically safer alternative.

BioID was developed as an alternative method, which involves the biotinylation of vicinal proteins using a mutated BirA, a DNA-binding biotin protein ligase. The advantage of BioID is that its function is not dependent on H_2_O_2_, making it safer than APEX. However, BioID’s labeling speed is relatively slow, taking 18–24 h to complete the labeling process, which may not capture quickly passing-by proteins (Roux et al., 2012). To address this limitation, TurboID was introduced. It operates on the same principle as BioID but offers quicker labeling and higher signal intensity, providing a balance between sensitivity and speed (Branon et al., 2018).

Despite the advantages of BioID and TurboID in terms of safety, they still lag behind APEX2 in terms of labeling speed. APEX2 is capable of accomplishing labeling in less than 1 min (Lam et al., 2015). Moreover, a recently developed light-activated proximity-dependent RNA labeling method has been introduced, which significantly reduces the labeling time to 0.6 ms. This method relies on miniSOG, an enzyme that can generate reactive oxygen species such as singlet oxygen and superoxide upon visible light illumination (Wang et al., 2019). It is worth noting that this method can also be adapted for protein labeling (Ren et al., 2023).

Indeed, the size of the tagging molecule and the labeling range are crucial factors that must be carefully considered when using proximity labeling methods. The tag size, such as that of APEX (57.401 kDa) and BirA (35.312 kDa), can potentially lead to disturbances for certain proteins with similar molecular weights. Moreover, the distance from the bait protein within which tagging occurs is also a critical consideration. APEX has a relatively larger labeling radius of about 20 nm, BioID has a range of approximately 10 nm (Christopher et al., 2021), and miniSOG has a substantial labeling range of about 70 nm (Wang et al., 2019). Since the components of MLOs can shuttle dynamically, and contaminants near the MLOs may inadvertently be labeled, the labeling range plays a significant role in distinguishing true interactors from nonspecifically labeled proteins. As a result, striking a balance between labeling speed and the labeling radius is of utmost importance.

Proximity labeling has been used to identify the constituents of various MLOs. For instance, both Apex2 (Markmiller et al., 2018) and BioID (Youn et al., 2018) have been employed to elucidate the composition of SGs, while BioID has been utilized to investigate the composition of PBs (Youn et al., 2018).

#### Other methods

In addition to the commonly mentioned methods in the previous section, there are also some less frequently utilized techniques that can offer distinct advantages. For example, Joseph Dopie et al. (2020) employed tyramide signal amplification MS (TSA-MS) to investigate the components of nuclear speckles. This method predates Apex-based proximity labeling and allows for the labeling of both the target protein and other nearby proteins after cells have been fixed and the target protein has been conjugated with its primary antibody. After conjugating horseradish peroxidase (HRP) to the primary antibody and adding H_2_O_2_, tyramide can bind to the tyrosine residues of the target protein and its neighboring proteins. Researchers have suggested that this method provides a broader labeling range compared to Apex-based labeling, typically falling within the range of 0.5–1 μM. However, it requires antibodies with exceptional specificity, and because labeling is performed after cell fixation, it may miss some dynamically interacting proteins with the target proteins. Moreover, apart from its wider labeling range, this method can act as a substitute for conventional proximity labeling methods when the marker for the specific MLO is too large for constructing plasmids that can link the sequence of marker to the cDNA of the tool enzyme. This is especially pertinent for crucial marker proteins found in nuclear speckles, such as SRRM2 and SON, which have molecular weights ranging from approximately 250 kDa to 300 kDa (Dopie et al., 2020).

In addition, due to the scaffold of paraspeckles being comprised of LncRNAs, specifically NEAT1_2 (Wang and Chen, 2020), it presents a challenge when attempting to attach enzymes such as APEX2 or BioID to the RNA. Nevertheless, researchers have explored alternative methods to analyze its components. West et al. (2014) employed MS to analyze CHART (capture hybridization analysis of RNA targets)-enriched material (CHART-MS) to identify proteins that interact with NEAT1 *in vivo*. This was accomplished by designing complementary oligodeoxyribonucleotides (CO) tagged with biotin, which could recognize specific LncRNA through base pair complementarity (Simon et al., 2011). Furthermore, the components of nuclear stress bodies were also analyzed using similar protocols (Ninomiya et al., 2020). Their formation is also driven by LncRNAs, specifically HSAT Ⅲ. However, in the case of this study, researchers have only provided information about the components of nuclear stress bodies after a 1-h recovery period following thermal stress. It is worth noting that the composition of these nuclear stress bodies may undergo changes both before and after the recovery period.

It is imperative to integrate the findings from both biochemical fractionation and proximity labeling methods when attempting to elucidate the constituents of MLOs. These two approaches possess distinct advantages and limitations, and by amalgamating their outcomes, a more accurate portrayal of the MLO’s component proteins may be achieved. However, the feasibility of applying these two kinds of methods should be considered based on different characteristics of different MLOs (Fig. 3).

Furthermore, MS analysis serves as a powerful tool for identifying potential components of MLOs based on their mass and charge. However, as previously emphasized, confirming the presence of these components within the MLO requires additional validation. Techniques such as immunofluorescence provide visual confirmation of protein localization within the organelle, thus substantiating the accuracy of MS-based findings. This traditional method was applied before the invention of proximity labeling, which was named localization screening, which means subcloning the genes of different proteins from a specific gene library into vectors containing fluorophores. Subsequently, these plasmids were overexpressed in cells, enabling the observation of the colocalization of specific proteins with markers associated with various MLOs (Fong et al., 2013; Naganuma et al., 2012).

### Analyzing common components between stress granules and other MLOs

**Figure 4.**
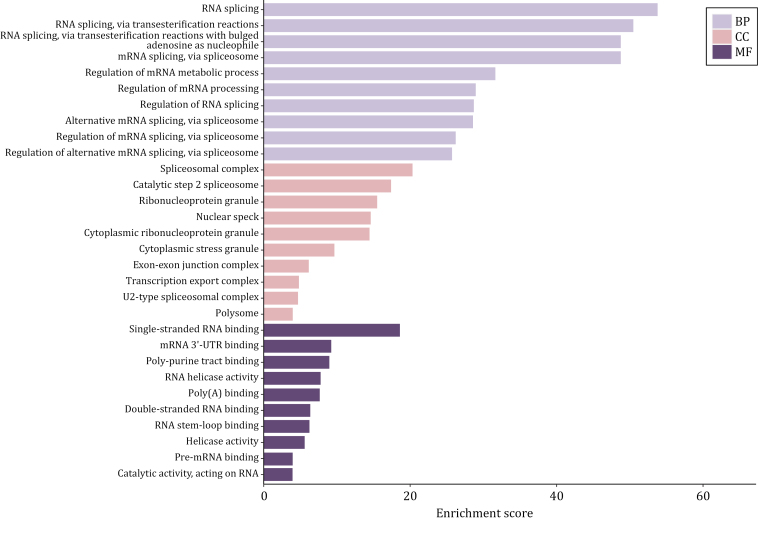
GO results of three ontologies of overlapped components of SGs and nuclear speckles. The overlapped components of SGs and nuclear speckles mainly function in RNA splicing and regulation of RNA splicing (created with bioinformatics).

**Figure 5.**
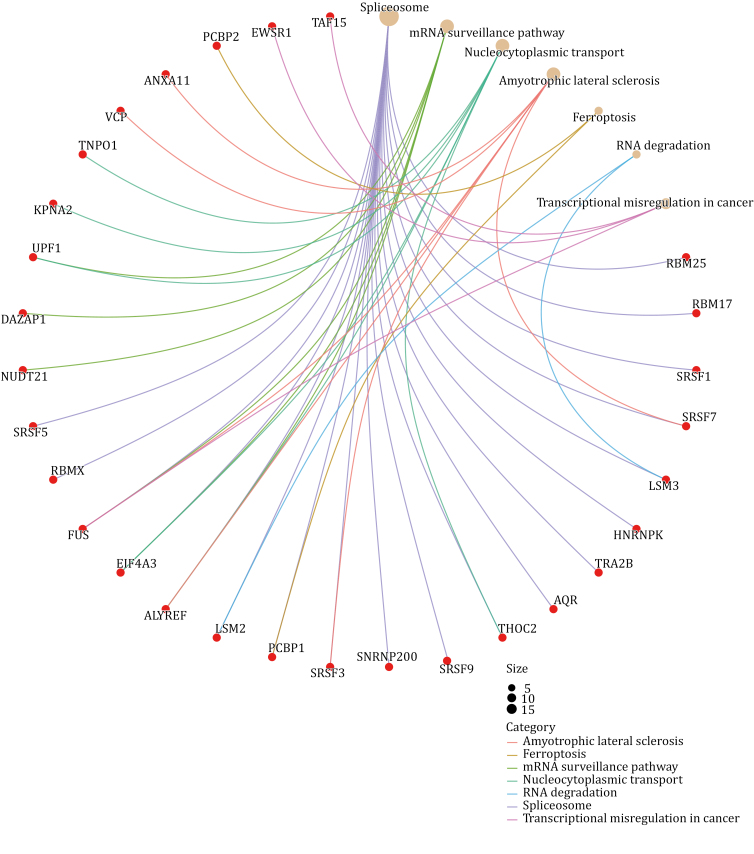
KEGG analysis of overlapped components of SGs and nuclear speckles. The overlapped components of SGs and nuclear speckles are associated with the diseases pathway of ALS (created with bioinformatics).

**Figure 6.**
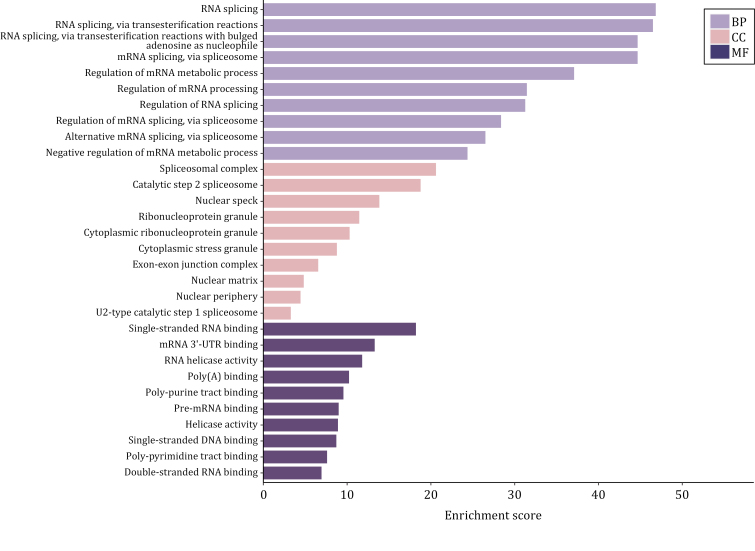
GO results of three ontologies of overlapped components of SGs and nuclear stress bodies. The overlapped components of SGs and nuclear stress bodies mainly function in mRNA processing and the regulation of mRNA metabolic processes (created with bioinformatics).

**Figure 7.**
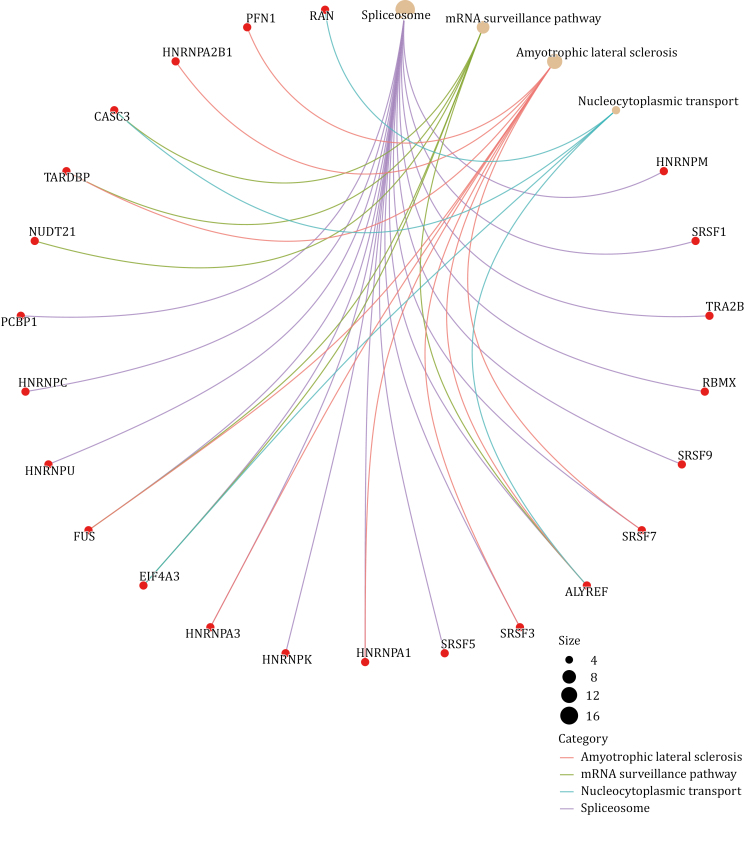
KEGG analysis of overlapped components of SGs and nuclear stress bodies. The overlapped components of SGs and nuclear stress bodies are associated with the diseases pathway of ALS (created with bioinformatics).

Focused on SGs, we specifically analyze their components in comparison with two other MLOs—nuclear speckles and nuclear stress bodies serving as an illustrative example. To begin, we retrieved the SGs components from RNA Granule Database Version 2.0 and specifically selected components classified as Tier 1, as these components are considered the gold standard for SGs components, resulting in a total of 473 members (Millar et al., 2023). As for the components of nuclear speckles and nuclear stress bodies, we reviewed relevant literature that aimed to identify these components using techniques, such as biochemical fractionation, proximity labeling, or other non-conventional methods (Table S1). Subsequently, we combined the elements from the various lists we obtained and conducted a GO analysis on these overlapping components. A total of 70 proteins are shared between nuclear speckle and stress granule, and the results of GO analysis indicate that the overlapping components primarily function in RNA splicing and regulation of RNA splicing (Fig. 4). Notably, these proteins show a high association with the pathogenesis of ALS by KEGG analysis (Fig. 5). Regarding nuclear stress bodies, there are 56 proteins in the overlapping region. GO analysis reveals that these components are primarily involved in mRNA processing and the regulation of mRNA metabolic processes (Fig. 6). Similarly, KEGG analysis indicates that the overlapping components are closely related to the pathogenesis of ALS (Fig. 7). The results highlight that the shared components primarily play a crucial role in regulating RNA metabolism and the pathogenesis of ALS. However, the study of RNA and the involvement of MLOs in RNA metabolism regulation remain challenging and require further researches.

### Methods to determine the role of shared proteins in regulating MLOs

In addition to comparing the overlapping components of different MLOs, it is crucial to investigate whether and when these shared components travel between different MLOs. A recent development by Qin et al. (2023) introduced an alternative method to track protein movement within the cell, effectively addressing this issue. This method not only verifies the presence of shared components between the two MLOs but also offers an alternative approach beyond the simple comparison of overlapping components. This method, known as Transit ID, combines TurbolID proximity labeling and Apex proximity labeling with Click reactions in a series. It allows proteins traveling from one location to another to be labeled with both biotin and Fluorescein. Using this approach, researchers obtained proteomic data on proteins moving between the nucleolus and SGs during stress induction and recovery (Qin et al., 2023). By leveraging this method, researchers can compile lists of proteins that transit between different MLOs, providing a more comprehensive view than simply comparing overlapping components. However, it is essential to confirm whether these transiting proteins change their position before and after stress through immunofluorescence.

After identifying potential components that may regulate MLOs, it becomes crucial to determine whether these components truly impact the behavior of MLOs. To ascertain this, one effective approach is knocking down specific components and observing the resulting changes in various MLOs. Recent research have applied siRNA screening and systematically recordings morphological changes of different MLOs to quickly screen out meaningful genes. By this method, researchers found that the depletion of the scaffold protein SRRM2 of nuclear speckle can induce the formation of SGs in a subset of cell in the absence of stress treatment, which were been illustrated before (Berchtold et al., 2018).

In addition, recent research has utilized CRISPR-Cas9 screening to examine the function of different RBPs in SGs assembly. This method can also be employed to assess the impact of shared components on various MLOs (Wheeler et al., 2020). Moreover, drug screening has proven to be highly valuable. In a study conducted by Wippich et al., chemical compound inhibitors were applied along with high-content imaging techniques to elucidate how these drugs impact the disassembly of SGs. Within these drugs, inhibitors of DYRK family kinases, such as DYRK3, are identified to delay the dissolution of SGs (Wippich et al., 2013). Furthermore, in subsequent years, researchers discovered that DYRK3 also plays a role in regulating the disassembly of other MLOs during mitosis (Rai et al., 2018). Therefore, image-based drug screening holds significant importance in studying how shared components can influence SGs or other MLOs.

## Discussion

This review highlights that SGs share numerous components with various organelles. Researchers have applied diverse methods, including biochemical fractionation, proximity labeling, CHART-MS, and FAPS to identify the components of MLOs. However, for some organelles like Cajal bodies and Gems, as well as newly discovered ones, there is still a lack of comprehensive understanding regarding their components. This calls for the application of established methods to further explore these organelles. Moreover, the composition of certain MLOs can change over time, particularly after drug treatments. For example, during heat shock, the components of SGs can vary at different treatment durations (Hu et al., 2023). In addition, different types of a specific MLOs may coexist, such as nuclear stress bodies (Aly et al., 2019). Hence, further research is essential to accurately elucidate the components and dynamics of diverse MLOs, leading to a deeper understanding of their roles.

While siRNA screening has been used to analyze the role of various genes in regulating MLOs, CRISPR-Cas9 screening has not been similarly applied, despite its potential for better-targeted gene studies. In addition, due to the sensitivity of MLOs to stress conditions, image-based drug screening could be valuable for studying drug impacts and enhancing our understanding of organelle interactions. Furthermore, given the sensitivity of various MLOs to distinct stress conditions, image-based drug screening can be valuable in studying the impact of drug treatment on different organelles. To gain a comprehensive understanding of the interplay between different MLOs, controlling one organelle and observing changes in another is critical. In this context, optogenetic induction of specific MLOs is a promising tool. It allows the emulation of stress-induced MLOs (Zhang et al., 2019). This controlled approach enables the observation of alterations in other biological condensates within cells. This refined approach offers a simplified model for studying the complex interactions between diverse MLOs.

To uncover the function of SGs, researchers have extensively studied their interactions with other organelles. These investigations highlight the collective response of different organelles to stress, underscoring the need for an integrative approach to understanding SGs beyond isolated examination. SGs, as stress-induced structures, recruit not only core components or scaffold proteins but also RBPs from various cellular locations during stress conditions. These proteins may originate from other MLOs or membrane-bound organelles. The processes involved in the recruitment of these proteins to SGs and their subsequent release are not yet fully understood. Furthermore, the significance of this dynamic relocation remains incompletely defined.

The concentration of RBPs in SGs and other RNP granules indicates a broader role of RNP organelles in RNA metabolism. They influence the localization and availability of RBPs, thus directly affecting RNA metabolism. For instance, recent research has shown that Quaking proteins, which bind to internally m7G-modified mRNAs, are recruited to SGs under stress conditions. This recruitment inhibits the translation of these mRNAs (Zhao et al., 2023). Consequently, it is hypothesized that shared RBPs transitioning between SGs and other MLOs under various stress conditions can provide valuable insights into the regulatory functions of SGs in RNA metabolism.

Beyond MLOs, SGs can also interact with other membrane-bound organelles or complexes. It has been observed that SGs can influence these organelles by recruiting their components during stress conditions or through their liquid droplet-like physical properties. Conversely, membrane-bound organelles and complexes can affect the dynamics and transport of SGs. However, further rigorous researches are needed to thoroughly validate these interactions. In this regard, super-resolution microscopy techniques are invaluable, as they allow researchers to examine the detailed structure of physical interactions between SGs and other organelles (Guo and Zhang, 2024).

In conclusion, understanding the functions and dynamics of SGs requires a comprehensive perspective that considers their interactions with both membraneless and membrane-bound organelles. By elucidating these interactions and the mechanisms of protein recruitment and relocalization, we can gain deeper insights into the roles SGs play in cellular stress responses and RNA metabolism. Future researches should focus on these integrative studies to fully comprehend the complex biology of SGs and their implications for cellular physiology and diseases.

## Supplementary data

Supplementary data is available at *Protein & Cell* online at https://doi.org/10.1093/procel/pwae057.

pwae057_suppl_Supplementary_Table_S1
